# Supplementary material for: Advancements in Life Tables Applied to Integrated Pest Management with an Emphasis on Two-Sex Life Tables
Source: Insects. 2025 Mar 3;16(3):261. doi: 10.3390/insects16030261 (PMC11943316; doi:10.3390/insects16030261)
Supplement: Supplementary file 1 [file insects-16-00261-s001.zip › insects-3458933-supplementary.pdf]

Table S1. Summary of research on the application of life tables in integrated pest control

Species	Key Findings	References
Orius strigicollis	Significantly increased feeding capabilities when fed 10 eggs. Highest R_0 and *GRR* achieved. Higher and similar *r* when fed 10 and 15 eggs due to higher fecundity and shorter/faster development times.	[**Error! Reference source not found.**]
Orius strigicollis	Type II functional response exhibited for both preys. Shorter prey handling time when fed *T. vaporariorum*. Shorter nymphal development and pre-oviposition period when fed *B. tabaci*.	[**Error! Reference source not found.**]
Amblyseius orientalis	Highest consumption rate, shortest development time, and highest cumulative fecundity. the r_m of 0.12. Sole species among tested phytoseiid mites expected to experience population growth when fed *P. latus*.	[**Error! Reference source not found.**]
Sitotroga cerealella	Higher values of λ, r_m, and R_0 when reared on maize. *T. chilonis* exhibited higher parasitism, adult emergence, longevity, and total adult longevity on *S. cerealella* eggs reared on maize. Consistent with evolutionary models, maize supports a higher proportion of female offspring in *T. chilonis*.	[**Error! Reference source not found.**]
Trichogramma euproctidis	Peak parasitism rates, highest number of female offspring, and greatest survival rates at 32.5 °C. Adapted to high temperatures and harsh environmental conditions.	[**Error! Reference source not found.**]
Tetrastichus howardi	The R_0 of 13.6 and *r* of 0.124. Mean generation time of 20.9 days. Natal host (*T. molitor* or *P. xylostella*) does not affect fitness or parasitism rate.	[**Error! Reference source not found.**]
Coccinella septempunctata	No significant side effects on the performance or biology of *C. septempunctata*. Non-persistent impact of entomopathogenic fungi on pest control.	[**Error! Reference source not found.**]
Culex pipiens	*M. anisopliae* showed the highest larval mortality (88%) and shortest LT_{50} (22.6 h). Reduced female fecundity, number of hatched eggs, pupation percentage, and adult emergence percentage. Changes in biochemical indicators.	[**Error! Reference source not found.**]
Spodoptera exigua	Significant impact on F_0 generation with cascading effects on F_1 demographic parameters. Decreased *r*, extended *T*, and reduced R_0 in F_1 offspring. Notably lower fecundity in infected groups compared to controls.	[**Error! Reference source not found.**]
Habrobracon hebetor	Low concentrations of HearNPV (LC_{30}) exert negative sublethal effects on *H. hebetor*, including	[**Error! Reference**

	reduced longevity and fecundity. Population growth parameters decrease with increasing virus concentration. In field conditions, *H. hebetor* can still effectively control *H. armigera* when released 2 days after HearNPV application.	**source not found.**]
Melanaphis sorghi	Estimation of expected lifespan based on biological parameters and fecundity life table. Identification of sorghum varieties unfavorable to the growth and reproduction of *M. sorghi.*	[**Error! Reference source not found.**]
Toxoptera aurantii	Analysis of endowment growth rate (*r*), net reproduction rate (R_0), and total lifespan (*T*) on different tea tree varieties. Variations in population dynamics and host adaptation among different varieties.	[**Error! Reference source not found.**]
Anopheles balabacensis	Life table analysis provides better estimates of mosquito survival rates. Survival rate estimates help assess the duration and likelihood of parasite development in mosquitoes and its transmission to secondary cases.	[**Error! Reference source not found.**]
Anopheles stephensi	Studied mosquito population growth and survival in different water analyzed how changes in water quality affect mosquito survival and vector competence.	[**Error! Reference source not found.**]
Culex quinquefasciatus and *Culex pipiens*	Pseudostage structured population dynamics model was employed. Photoperiodicity and temperature are critical factors influencing larval stage duration. Life history observations under natural field settings can accurately predict insect development across the annual cycle.	[**Error! Reference source not found.**]
Whitefly vector	Developed a temperature-dependent phenology model using ILCYM software. Transmission capacity of whitefly was strongest at 15 °C (70% infection probability) but dropped sharply at 10°C and 20°C. A comprehensive temperature-responsive model was created to predict the spread potential and transmission probabilities of PYVV. Risk maps were generated using the best-performing risk index, accurately reflecting virus occurrence and predicting high-risk areas.	[**Error! Reference source not found.**].
Anopheles coluzzii	The bacterium effectively infects *A. coluzzii* larvae. Strain IRSSSOUMB001 shows pronounced virulence against insecticide-resistant *A. coluzzii* larvae. Decreased mosquito fecundity and fitness of progeny due to bacterial infection.	[**Error! Reference source not found.**]

Aedes aegypti	Mosquitoes depend on key nutrients at multiple life stages. Targeting metabolic pathways can disrupt mosquito development and infection. Alginase inhibitors are suggested as potential next-generation insecticides.	**[Error! Reference source not found.]**
Phenacoccus solenopsis	All tested host plants were suitable for *P. solenopsis.* Eggplant exhibited the highest fecundity, net reproductive rate (R_0), finite rate of increase (λ), and longest adult longevity (males: 6.50 ± 0.34 days; females: 24.15 ± 0.50 days).	**[Error! Reference source not found.]**
Acanthococcus lagerstroemiae	Higher intrinsic rate of increase, finite rate of increase, and net reproductive rate under nutrient-deficient conditions. Longer mean generation time under nutrient-rich conditions. Better performance on plants under nutrient-deficient conditions.	**[Error! Reference source not found.]**
Aphis gossypii Glover	Imidacloprid-resistant lines (ImR) exhibited prolonged developmental stages, shortened longevity, and decreased fecundity. Significant reduction in key demographic parameters in ImR, indicating a fitness cost associated with resistance. Molecular changes: altered expression of genes related to development and reproduction, with some genes downregulated.	**[Error! Reference source not found.]**
Spodoptera frugiperda	Fitness cost associated with chlorpyrifos resistance observed at both individual and population levels. Life table parameters indicated reduced fitness in resistant populations. Potential for resistance levels to decrease with the withdrawal of selective agents from the environment.	**[Error! Reference source not found.]**
Laodelphax striatellus	LC_{50} of triflumizole-pyrimidines significantly reduced the intrinsic rate of increase (r), finite rate (λ), and net reproductive rate (R_0) in the F_5 generation compared to F_0. Triflumizole-pyrimidines may impede generational growth and reproduction of *L. striatellus.*	**[Error! Reference source not found.]**
Bradysia odoriphaga and *Bradysia difformis*	Dinotefuran and lufenuron were highly toxic to *B. odoriphaga* and *B. difformis* compared to other tested pesticides. Sublethal doses of these pesticides significantly impaired life-history parameters and detoxification enzyme functions in both generations.	**[Error! Reference source not found.]**
Hypothenemus hampei	life table constructed by calculating survival and reproduction rates across developmental stages. Net reproductive rate (R_0) determined for each population. Optimal timing for pest control interventions identified.	**[Error! Reference source not found.]**
Halyomorpha halys	Comprehensive study combining laboratory and field experiments. Influence of temperature fluctuations	**[Error! Reference**

	on life stages of *H. halys* examined in diverse geographical regions within the United States. Quantified patterns of key parameters at different temperature levels.	**source not found.**]
